# Supplementary material for: Role of Polymeric Coating on Metallic Foams to Control the Aeroacoustic Noise Reduction of Airfoils with Permeable Trailing Edges
Source: Materials (Basel). 2019 Apr 2;12(7):1087. doi: 10.3390/ma12071087 (PMC6480125; doi:10.3390/ma12071087)
Supplement: Supplementary file 1 [file materials-12-01087-s001.pdf]

Supplementary Materials: Role of Polymeric Coating on Metallic Foams to Control the Aeroacoustic Noise Reduction of Airfoils with Permeable Trailing Edges

Reza Hedayati [1,*], Alejandro Rubio Carpio [2], Salil Luesutthiviboon [2], Daniele Ragni [3], Francesco Avallone [3], Damiano Casalino [3] and Sybrand van der Zwaag [1]

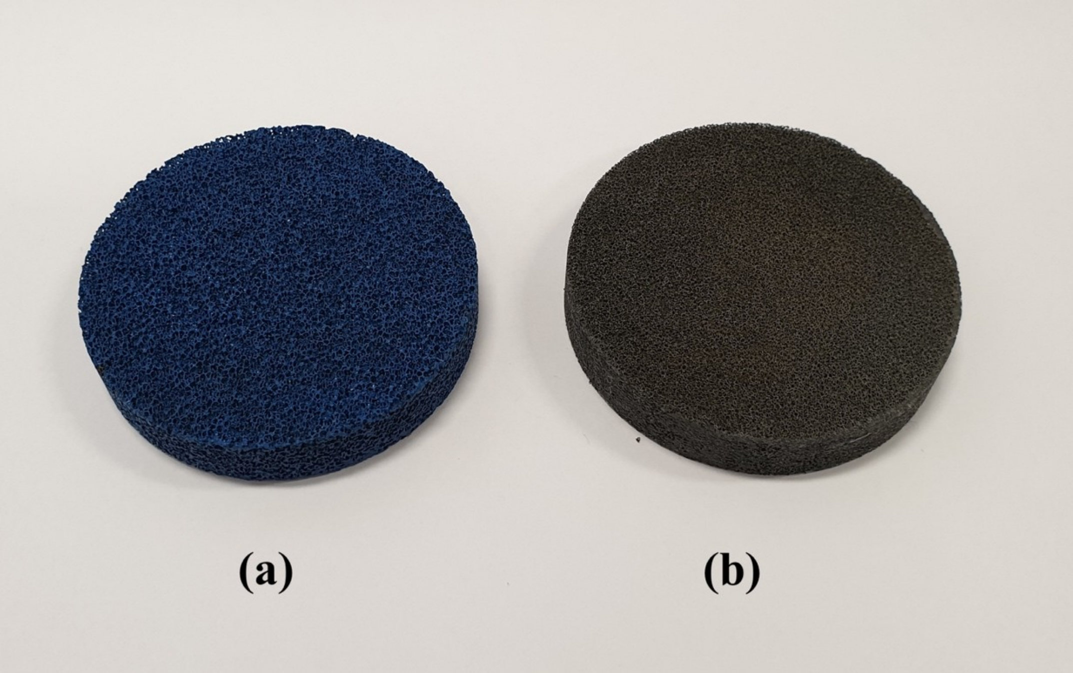

Figure S1. Examples of disks used for characterization of the permeability of (**a**) coated and (**b**) uncoated metal foams.
